# Toward adapting the UN’s healthy aging agenda for India: tailoring to unique historical context and traditions

**DOI:** 10.3389/fpubh.2023.1346962

**Published:** 2024-01-11

**Authors:** Nandakumar Bidare Sastry, Monika Vempadapu, Shalini Sivananjiah

**Affiliations:** ^1^Research and Innovation, Ramaiah University of Applied Sciences, M S Ramaiah University of Applied Sciences, Bangalore, India; ^2^Division of Research and Patents, Community Medicine, Ramaiah Medical College and Hospitals, M S Ramaiah University of Applied Sciences, Bangalore, India; ^3^Division of Research and Patents, Ramaiah Medical College, M S Ramaiah University of Applied Sciences, Bangalore, India; ^4^Department of Community Medicine, Ramaiah Medical College, Ramaiah International Medical School, M S Ramaiah University of Applied Sciences, Bangalore, India

**Keywords:** older adult, healthy aging, healthcare, healthcare services, National Health Programs, digital health mission, India

## Abstract

India is known for its rich cultural heritage with different cultures and customs. Indian historical traditions and cultures were molded in a manner that most older adults were cared for at home by their children. However, India is being urbanized and developing swiftly changing its socio-cultural scenarios. With globalization and the increased popularity of social media, the youth is more ambitious than ever and is ready to migrate and explore. Fueled by the rapid aging of the global population, demand is escalating for robust programs, policies, and activities to improve the lives of older adults. However, most of these schemes have not yet been fully implemented nationwide; several state governments have yet to realize their full potential due to the lack of resources and competing priorities. Aligning with the UN’s healthy aging agenda, several programs and policies in India are contributing toward ensuring quality aged care services. This paper explores the challenges and opportunities for effective ground-level translation from precepts to practice.

## Introduction

India is known for its rich cultural heritage with different cultures, religions, customs, traditions, and languages. Thus, the Indian social matrix and the cultural pattern are characterized by “Unity in Diversity” (1). Many mythological stories show that the aged parents were respected and worshipped like Gods, as in the story of Shravan Kumar, known for his filial piety toward his parents (2). *Thaittiriya Upanishad* states “Let your mother be God. Let your father be God” which brings out the importance of caring for older adults which is deeply rooted in the traditions (3). Indian historical traditions and cultures were molded for the care of an older adult at home by their children. Also, older adults have a societal expectation from their children, a tradition that has been long-standing and pervasive (4).

India is rapidly urbanizing and developing swiftly with changing socio-cultural scenarios. With globalization, the youth is ambitious to migrate and explore (5). There is also an increased participation of women in the workforce (6). These factors have caused a shift from the idea of a joint family to a nuclear family over time leaving the older adults to look after themselves. This is compounded by the system’s failure to offer adequate pension and healthcare services for older adults (7).

In this perspective article, we offer our viewpoints on how India is tailoring its unique cultural and historical traditions to conform with the guidelines and expectations of the UN’s Healthy Ageing Agenda.

## Epidemiology of aged care in India

The population of older adults in India was 104 million (8.6% of the total population) according to the 2011 census, and is projected to increase to 173 million by 2026 (8). According to the World Health Organization (WHO), one in six people in the world will be aged 60 years or older by the year 2030. Alongside the rapid aging of the global population, demand is escalating for robust programs, policies, and activities to improve the lives of older adults. Therefore, today it is essential to increase the focus on providing healthy life expectancy of the population.

## Policies and programs

Over the years, India proposed and implemented policies, programs, and legislation for the older adults. The government has formulated policies and legislation to facilitate the care for the older adults within the families, thereby tailoring to the cultural and historical context in India. Since 1992, Government of India has implemented various schemes and programs, through different Ministries and Departments for care and welfare of senior citizens (Table 1). However, most of these schemes have not yet been implemented nationwide; many state-level governments have failed due to lack of resources and competing priorities.

**Table 1:** Policies, agenda, and implementation challenges in India.

Schemes	Agenda	Challenges
National Policy for Older Persons, 1999	Pensions, travel concessions, income tax relief, medical benefits, extra interest on savings, and security of older persons through an integrated scheme of the Ministry of Social Justice and Empowerment	Absence of savings. Physical decline Increased Destitution
Indira Gandhi National Old Age Pension Scheme, 1995	Old age pension scheme would cover all senior citizens living below the poverty line. The rate of monthly pension would be raised to Rs.1000 per month per person and revised at intervals to prevent its deflation due to the higher cost of purchasing.	The monthly pension is not proportionate to the rate of inflation. Poor and irregular flow of funds will add to the plight of elders
The Maintenance and Welfare of Parents and Senior Citizens Act, 2007	Public distribution system under Annapurna scheme to provide free rice up to 10 kgs for destitute	Lack of awareness, supply chain and consumer availability
National Policy for Senior Citizens, 2011	The policy seeks to reach out to the bulk of senior citizens living in rural areas who are dependent on family bonds and intergenerational understanding and support. The policy will consider institutional care as the last resort.	Family migration to urban areas for better job opportunities and resistance of elders to adapt to the new environment.
National Programme for Healthcare for the Older Adults (NPHCE), 2011	To provide accessible, affordable, and high-quality long-term, comprehensive, and dedicated care services to an aging population	Urgent need for developing specialized health services for older people at primary, secondary and tertiary care levels given their rapidly increasing number with varied health, economic and psycho-social needs. Outreach services for patients discharged for follow-up care. Multidisciplinary team to provide comprehensive services
Central Sector Scheme of Integrated Programme for Senior Citizens (IPSrC), 1992	Grants in aid are given for running and maintenance of Senior Citizens Homes (Old Age Homes)/Continuous Care Homes, Mobile Medicare Units	Registration of Senior Citizens Homes, poor infrastructure and maintenance, lack of funds, lack of trained caregivers, non-admission of elders with poor ADL, nonfamilial support
Rashtriya Vayoshri Yojana (RVY), 2017	Aids and assistive living devices are provided to senior citizens belonging to the BPL category or those senior citizens who earn less than 15,000/− per month and suffer from age-related disabilities	Identification of beneficiaries and customized good quality supply of appliances is a challenge. Lack of awareness by older adults adds to the low usage and distribution
National Helpline for Senior Citizen, 2021 Elder line (14567)	The helpline is to address the grievances of the elders.	Inability, lack of awareness and underreporting of elder abuse, security
State Action Plan for Senior Citizens (SAPSrC)	Each State/UT is expected to plan and strategize taking into account their local considerations and frame their own State Action Plans for the welfare of their senior citizens. This Plan takes care of the top four needs of the senior citizens *viz.* financial security, food, health care and human interaction /life of dignity	Challenges in trained manpower, creation of multidisciplinary teams and capacity building will be visible. Funds for Infrastructural facilities may be delayed.
SAGE, 2021	Innovative start-ups will be identified and encouraged to develop products, processes, and services for the welfare of the older adults under this initiative	It may be difficult for committed youths to work for this initiative.
Atal Vayo Abhyuday Yojana, 2021	Awareness generation/sensitization programs with school/college students for strengthening Inter-generational bonding.	There is a disintegration of joint family and the concept of one child norm with both working parents. Physical distance between grandparents and only one grandparent alive is also another challenge.

## Need for quality care for older adults in India

Beyond expanding and strengthening networks of health care provision for older adults, a need and an opportunity exist to utilize technology to aid people living in remote areas, or for those who have limited mobility. One promising technology application is information and resource ‘telehealth’ call and monitoring centers that offers health advice and support to older adults (9).

Both low-tech and high-tech innovations are also needed to help maintain older individuals’ independence, dignity, and quality of life; low-cost and readily available versions of adaptive devices and aids, such as walkers, hearing aids, reading glasses and magnifiers, and grab bars present another form of technology solution. Additionally, infrastructure in India needs to be more accommodating for older adults and those with disabilities. As India continues to modernize its infrastructure, designing spaces for an aging population will involve building structures, adapting transportation, and implementing services that meet the needs of older adults and address the principles of inclusivity, accessibility, and connectivity (9).

## UN healthy aging agenda

In 1994, the World Bank saw population aging as a crisis that needed ‘averting’ (10). Ensuring the healthy aging of older adults has been at the forefront of the UN agenda since 1982, with the formulation of the Vienna International Plan of Action on Ageing (11, 12). Despite collective achievements in recognizing and advancing the rights of older adults, the pressing need for improving the health and well-being of older persons was not central in the conception of the multilateral 2030 Agenda for Sustainable Development.

**Figure 1:**
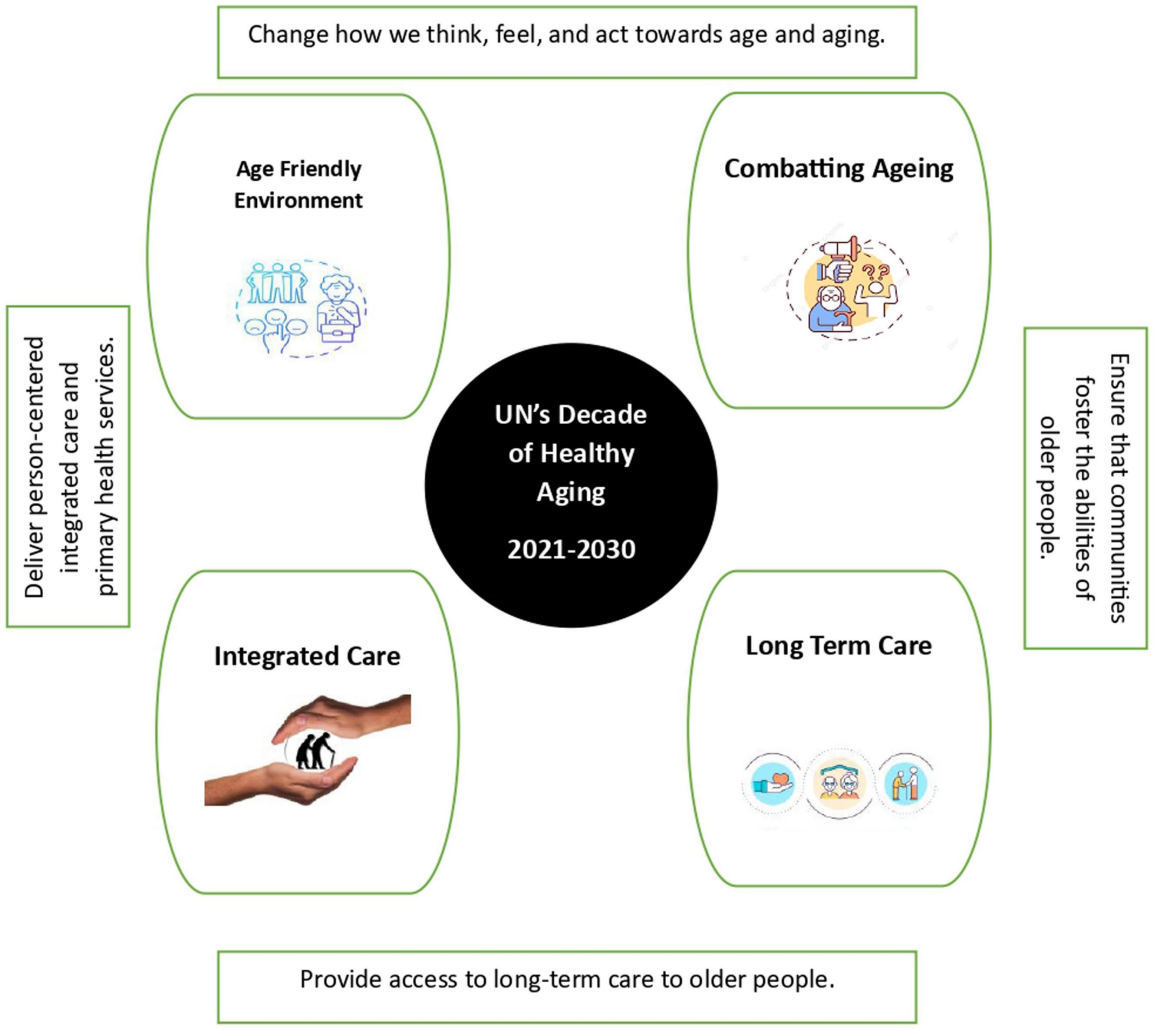
Key initiatives to foster healthy aging.

To foster healthy aging and improve the lives of the older adults, their families and communities, fundamental shifts will be necessary both in thoughts and actions. The 2021–2030 UN’s Healthy ageing addresses four action areas (Figure 1).

*Age friendly environment*: Some of the prerequisites for age friendly environment are: room in the ground floor with switches close to the bed, commode with rails and antiskid bathroom without pooled lighting, ramp if needed to go upstairs, uncluttered areas for movement. Outside the home, roads should be well asphalted with even surfaces, visual and sound prompts and signals for lane intersection for senior adults with various handicaps. Structures are well-set apart outside and inside, with satisfactory seating and toilets, accessible lifts, slants, railings and stairs, and nonslip floors (13).

*Combating aging*: or “healthy aging” encompasses regular exercise and physical activity, making smart food choices, relaxing bedtime with 8–9 h of sleep, annual health checkup and more frequently, if symptoms develop. Healthy aging also includes being socially connected with friends and relatives, practicing mindfulness and reading books, pursuing hobbies (14).

*Integrated Care*: WHO has developed Integrated Care for Older People (ICOPE) to meet the demand of the increasing older population. The delivery models could be community-based or home-based interventions at organizational level or at the clinical level (15, 16). The assessment could be personalized, and care plans integrated into it. The decision-making between the older adult and the physician should be shared, goals need to be set and work toward it. The physician can assist in supporting self-management and provide linkages with social support services. At the management level, data should be shared, the community should be involved and support provided for caregivers (17). The ultimate aim is to maximize the intrinsic capacity and functional ability of the older adults, with comprehensive assessments of all dimensions (Physical, Mental and Social Well-being) and not morbidity centric approach alone.

*Long Term Care*: It is the medical, and psychosocial support for older adults with limited Activities of Daily Living (ADL), Instrumental Activities of Daily Living (IADL), and chronic morbidities including palliative care. They need support from family, caregivers, community, or institutions. Caring for senior adults with mental instability, Parkinson’s disease and Alzheimer’s is a challenge for family members or paid caregivers. Tata Institute of Social Sciences (TISS) provides professional courses for social work for older adults in India. Social work professionals in the field are however very scarce (18).

## Issues, concerns, and solutions

There is pressure on the health system for age-appropriate health care especially long-term care. The services and technologies for prevention, and treatment of diseases has not been forthcoming. With the dwindling joint family system, household care support is becoming more challenging. Further, the insecurity of family income and even if older adults are a pensioner, this source may not be adequate to support for a paid caregiver. It is also observed that there is prejudice and discrimination for ageism. Though not adequately documented, financial abuse of older women and men due to property dispute among the siblings is observed. Indian elders are overwhelmed by the affection to their children; hence they turn a blind eye to the environment around them.

However, opportunities can be tapped with the available resources for income generation. There is scope for providing and expanding social protective measures. Jobs can be created for older adults with flexible working schemes with no limitation for working age. They could work online in marketing, communications, designing, voluntary social activities. Need based skills training can be provided. Those in the rural areas can be trained on paper making, food processing, agarbathi making, mushroom cultivation etc. Day care centers can be initiated for elders for social security.

*Aging in place*: Due to the breakdown of joint families, and the moving away of children for better job opportunities, older adults prefer aging in place because of familiar immediate surroundings and neighborhood. If they are physically independent, medically, and mentally fit, there are no issues. However, challenges emerge when older adults are left alone as the slow withdrawal in daily activities raises the risk of crime.

Older adults can be provided with personal security, contact cards can be displayed and friendly cops can ease the situation, as in the “SAVERA YOJANA” initiative by the Uttar Pradesh Police (19). Digital innovative solutions for older adults in urban areas can be developed by entrepreneurs to ensure safety and security, providing daily essential activities, home care, hygiene, and innovative health care. Additionally, empowering the family members and informal caregivers with certain skill will help the older adults to stay in residential environment rather than facility-based dwellings.

*Age-friendly environment*: Rented houses/apartments may not be conducive to modifications. In such instances, need-based temporary alterations may be a necessity. Several apartment builders are providing the option of minor customization to suit the requirements of older adult population within their projects. In India, there are ample opportunities for spiritual well-being through religious activities both within internal (domestic) and external (community) environment.

*Combatting aging*: In India, it is the psychosocial and financial security that needs to be addressed especially with migration, poor socioeconomic status, and lack of education contributing to the plight of older adults. Therefore, it is imperative to inform older adults about the maintenance and welfare of parents/senior citizens, the net of social safety measures available, and continued employment beyond the retirement age through Mahatma Gandhi National Rural Employment Guarantee Act (MNREGA). The measures to be undertaken by the policy makers/Government are periodic review of select social welfare schemes, revisiting its retirement and pension policy, removal of administrative bottlenecks and simplification of required documentation, enhancement in the pension amount, and adoption of a transparent disbursal mechanism.

Private health insurance does not cover the chronic morbidities in older adults though it is needed the most at these critical times of life. The premium escalates due to inflation and the coverage is after a waiting period of three to 4 years. If any untoward health event occurs during this period, it could be disastrous.

There are success stories on older adults’ self-groups in India which can be studied and implemented among those in the lower socioeconomic status and living in rural areas with community participation.

Food insecurity can be solved via the ICDS (Integrated Child Development Scheme) program in the Anganwadi, public distribution system by extending this to the older adults who are unable to cook. Meals on wheels at subsidized rates could also be encouraged.

*Integrated care*: This issue can be addressed through Community-based day care centers, NGO’s and Panchayat Raj Institutions, mobile medical clinics, and home-based care. The challenge for utilizing day care centers is that the older adult should have good ADL, IADL, accessibility and affordability to reach the day care center. Community based palliative care and dementia care are other explored areas with challenges of restricted trained and compassionate manpower.

*Long term care*: Practitioners are inadequately prepared to provide comprehensive care to the older adults. The informal caregivers and family caregivers are less prepared for these responsibilities and the burden of caregiving is translated to abuse of older adults (20). Equipping the family members to handle caring and daily decision-making of managing the chronic illness is a Herculean task, which should be addressed.

### Newer models of financing and care delivery

- **“Adopting a granny”** by school children especially when an old age home and school for orphans’ is cohabitated. This helps in intergenerational bonding and reduces the psychological stress. It also prevents burnout among the caregivers.
- Capacity building on caregiving, bedside assistance, and methods to improve intergenerational bonding under the aegis of NICE **(National Initiative on Care of Elderly)**
- Clinicians could utilize **digital innovations** for predicting falls, ensuring early interventions which in turn supports the healthcare system through reduced ICU (Intensive Care Unit) admissions and medical expenditure.
- Promoting **Silver economy** by the Government with cash assistance to encourage entrepreneurs to think tank on innovative solutions for older adults.
- **Corporate Social Responsibility (CSR)** funds by companies for older adults for infrastructure and or care is an approved item yet untapped resource.

### Adapting to Indian context

The number of older adults is bound to increase with better health affordability, improved technology, higher life expectancy and feminization, and evolving family structure both in urban and rural areas. It is therefore imperative that we organize our fragmented older adult care ecosystem. A multipronged approach is essential for acute and long-term care of older adults. India needs to evolve a framework with appropriate policies to attract investment in the sector, quality standards should be set for care delivery and appropriate timely regulations should be in place. Co-financing for the health of senior adults can mitigate health expenditure. For the home environment to be safe and secure for older adults, innovative cost effective, feasible digital tech solutions should be developed and nurtured by entrepreneurs.

**Case Study**

In southern India, Mrs. X, aged 80 years, a widow with three children (two males and one female), went into an institutionalized home due to the absence of a devoted full-time family caregiver. Her husband’s pension was the only financial support but could not meet the entire medical expenses. Philanthropic organizations helped her to deal with this (socio-cultural support was provided in the institutional setting). Since she was a resident for a long time, her last few days were spent peacefully in the same place. Finally, she donated her body to the medical college for the student’s learning.

Despite the availability of such philanthropic organizations and non-governmental agencies (NGO) many are not aware about them. Owning to the urban concentration of such facilities, the affordability to avail some of these services are beyond the reach of many older adult.

**Key Observations:**

- Inadequate knowledge about professional caregivers and the organizations providing such services.
- Lack of affordability and absence of pooled human and financial resources.
- Dealing with co-morbidities through an integrated multi-disciplinary approach.

**Proposed Direction of Travel:**

Ageing in place in the familiar environment of home through trained domiciliary care givers could be a potential solution.

## Data availability statement

The original contributions presented in the study are included in the article/supplementary material, further inquiries can be directed to the corresponding author.

## Author contributions

NS: Writing – original draft, Conceptualization, Formal analysis, Methodology, Supervision, Validation. MV: Conceptualization, Methodology, Writing – original draft. SS: Writing – review & editing, Conceptualization, Supervision.
